# Use of alternative consultation forms in Danish general practice in the initial phase of the COVID-19 pandemic – a qualitative study

**DOI:** 10.1186/s12875-021-01468-y

**Published:** 2021-06-02

**Authors:** Tina Drud Due, Thorkil Thorsen, Julie Høgsgaard Andersen

**Affiliations:** The Research Unit for General Practice and Section of General Practice, Department of Public Health, University of Copenhagen, Øster Farimagsgade 5, 1014 Copenhagen, Denmark

**Keywords:** General Practice, Primary care, COVID-19, Video consultation, Telephone consultation, Pandemic

## Abstract

**Background:**

Attempts to manage the COVID-19 pandemic have led to radical reorganisations of health care systems worldwide. General practitioners (GPs) provide the vast majority of patient care, and knowledge of their experiences with providing care for regular health issues during a pandemic is scarce. Hence, in a Danish context we explored how GPs experienced reorganising their work in an attempt to uphold sufficient patient care while contributing to minimizing the spread of COVID-19. Further, in relation to this, we examined what guided GPs’ choices between telephone, video and face-to-face consultations.

**Methods:**

This study consisted of qualitative interviews with 13 GPs. They were interviewed twice, approximately three months apart in the initial phase of the pandemic, and they took daily notes for 20 days. All interviews were audio recorded, transcribed, and inductively analysed.

**Results:**

The GPs re-organised their clinical work profoundly. Most consultations were converted to video or telephone, postponed or cancelled. The use of video first rose, but soon declined, once again replaced by an increased use of face-to-face consultations. When choosing between consultation forms, the GPs took into account the need to minimise the risk of COVID-19, the central guidelines, and their own preference for face-to-face consultations. There were variations over time and between the GPs regarding which health issues were dealt with by using video and/or the telephone. For some health issues, the GPs generally deemed it acceptable to use video or telephone, postpone or cancel appointments for a short term, and in a crisis situation. They experienced relational and technical limitations with video consultation, while diagnostic uncertainty was not regarded as a prominent issue

**Conclusion:**

This study demonstrates how the GPs experienced telephone and video consultations as being useful in a pandemic situation when face-to-face consultations had to be severely restricted. The GPs did, however, identify several limitations similar to those known in non-pandemic times. The weighing of pros and cons and their willingness to use these alternatives shifted and generally diminished when face-to-face consultations were once again deemed viable. In case of future pandemics, such alternatives seem valuable, at least for a short term.

## Background

Attempts to manage and mitigate the COVID-19 pandemic have led to radical reorganisations of societies and health care systems worldwide [1]. In Denmark, the first patient with COVID-19 was detected on February 27^th^, 2020. On March 11^th^, there were 514 confirmed cases and the government announced a lockdown of society to alleviate the spread [2]. Shortly after, the Danish National Board of Health published its recommendations on how to handle the epidemic in the Danish healthcare system. Non-critical activities across the health care sectors were put on hold to free resources for taking care of patients with COVID-19 and to prevent spreading the disease [3, 4].

General practitioners (GPs) are the first point of contact for patients with health concerns, and they provide the vast majority of patient care and treatment. Therefore, during the pandemic the GPs had to balance two needs: preventing the spread of COVID-19, while providing their patients with regular care for other health issues.

A financial agreement was made for general practice soon after the lockdown, enabling remuneration for the use of video and extended telephone consultations. In the middle of March 2020, The Danish College of General Practitioners (DSAM) published a so-called green-yellow list with guidelines for GPs, which suggested who to see in the clinic (yellow), and whom to offer a video or extended telephone consultation (green). Some examinations were suggested to be postponed [5]. The strategy for slowing down the spread of the disease worked, and from the middle of April, the rate of infection, hospitalization and death decreased substantially [6]. A re-opening of society began, and the health care system increased activities not related to COVID-19. In general practice, there was an increase in the number of health issues seen in the clinic [7].

Knowledge on how general practitioners have experienced handling the pandemic is scarce. With the pandemic still among us and a current widespread rise in COVID-19 cases, it is crucial to obtain knowledge on how the pandemic influences general practitioners’ possibilities to provide care for all the other, non-COVID-related health issues. Therefore, our study asked how Danish GPs experienced reorganising their work in their attempt to uphold adequate patient care while contributing to minimizing the spread of COVID-19. Further, and in relation to this, our study asked what guided GPs’ choices between telephone, video, and face-to-face consultations. This knowledge can be used when considering the pros and cons of reorganising consultation forms during the current pandemic, as well as in future pandemics that may require radical changes of medical work.

## Methods

### Design and data collection

We conducted a qualitative study involving 13 general practitioners. They were recruited through our colleagues at the Research Unit for General Practice in Copenhagen in order to make the recruitment process as fast as possible, so that we could obtain data from the initial phase of the pandemic. We went for 12–15 GPs as a relevant and manageable number. We recruited participants while interviewing, and after 13 participants we believed to have obtained a sufficient variation in selection criteria and experiences. We strove to reach variation among the participants in terms of practice type, gender and seniority. The participants all had clinics in either Region Zealand or The Capital Region. Three GPs had solo practices, while the rest were in partnership practices of differing sizes. Five participants were male. We interviewed the GPs twice. The first interview investigated the GPs’ initial responses to and experiences of the pandemic. The second interview examined how their experiences and responses had changed as the pandemic had developed, including how they had engaged in new consultation forms. JHA and TDD conducted the first round of interviews in the beginning of April 2020 and the second round of interviews with 11 of the GPs at the end of June. A twelfth GP was interviewed at the end of August, and the last declined a second interview. The interviews were conducted over video to avoid any risk of COVID-19 infection. The themes of the interview guides are presented in Table 1. To get an impression of how daily work in the clinic was affected by the pandemic, the participants were asked after the first interview to take written or audio recorded daily notes for 20 days, taking a point of departure in the four questions in Table 1. Eleven of the GPs provided notes. The GPs were remunerated for their participation in the data collection in accordance with the general agreement for general practice. This remuneration is customary and often a condition for the participation of Danish GPs in research projects because their income is based mainly on fee-for-service reimbursement.

**Table 1:** Interview guide and daily notes

Interview themes in the first interview
• Physical and organizational precautionary measures taken in the clinics to minimize the risk of spreading COVID-19
• Use, reasoning and experience with telephone and video consultations
• The GPs perceptions of their own risk of infection and their work conditions during the pandemic
• Tasks and challenges related to patients with COVID-19 symptoms
• Information from the authorities and keeping up-to-date
• Work and challenges related to implementing guidelines
• Collaboration with the rest of the health care system
Interview themes in the second interview
• Follow up on issues covered in the first interview and in the notes
• Continued reorganisation and resumption of usual practice
• Use, reasoning and experience with telephone and video consultations
• Experiences of clinical quality during the pandemic and adequacy of treatment for patients with COVID-19 symptoms and patients with regular health issues
• The influence of the minimal use of personal protective equipment on patient care
• Counselling patients in the increased risk of serious illness if infected with COVID-19
Questions for the written or audio recorded daily notes
• Which clinical challenges have you experienced today in relation to COVID-19 and other acute/sub-acute health issues?
• Are you aware of any changes in clinical guidelines since yesterday?
• Is there anything else that has taken up time today?
Do you have any suggestions as to how the situation in general practice could be improved?

### Analysis

We conducted an explorative and inductive analysis of the interviews. We started by reading the entire material upon which we decided on a coding structure. All authors then coded the same two interviews to compare our use of the codes and the coverage of our initial coding structure. This process resulted in a few changes of the coding structure. Each of us then coded a third of the remaining interviews using the software programme Nvivo 11 Plus. We then compared the themes and coded extracts both within and across interviews and wrote a coherent narrative of each theme. In case of questions or puzzlement, individual interviews were re-read. The notes from the GPs were not coded in Nvivo. Instead, we identified the main themes to see whether they differed significantly from the interviews, which they did not. The themes predominant in the interviews and related to the aim of this study are presented in Table 2.

**Table 2:** Themes from the analysis

• Reorganising consultations
• Choice between face-to-face and video/telephone consultations
∘ Minimization of risk
∘ Following guidelines
∘ A preference for face-to-face consultations
• Experienced differences between face-to-face and video/telephone consultations
∘ Consultation content and form
∘ Relational and nonverbal limitations
∘ The clinical assessment and treatment
∘ Technical limitations
∘ Choice between video and telephone consultation

### Ethics

All participants received written information about the project, signed an informed consent form and were promised anonymity. The data are securely stored and can only be accessed by the authors. The project has been registered with the Danish Data Protection Agency (journal nr. 514–0491/20–3000) and has been presented to the Danish ethics committees, which declared that being a qualitative study, it does not need their approval (journal nr. 20,023,269).

## Results

### Reorganising consultations

Following recommendations from The National Board of Health and DSAM, the GPs cancelled all consultations in the first days after the lockdown. However, after a few days they initiated new ways of practicing to handle their patients’ regular health care needs in the best way possible, given the risk of COVID. This implied that a part of their consultations was resumed as face-to-face consultations while others were converted into video or telephone consultations, or postponed or cancelled.

Most of the interviewed GPs expressed that it had been challenging planning which patients to see and how. Therefore, they had daily meetings in the clinics discussing this issue, and they made frequent changes, especially in the beginning.

*"In the beginning, it was almost every half day we made changes regarding who we were going to see. We have a large whiteboard where we have tried to outline which patients we should see. These type of patients - what are we going to do with them? And it has been corrected many, many times.” *(GP8, interview 1)

Examples of postponed or cancelled activities were chronic care check-ups (if the patient was usually well-regulated), paediatric examinations, and smear examinations. These were consultations needing physical attendance but deemed non-acute and postponable. Spirometry was not undertaken in any of the study clinics during this period (being non-acute and entailing too much of a risk for the staff), and some blood tests and electrocardiograms were suspended in the beginning but resumed during the study period. Patients with respiratory problems could not be seen in the clinics. At the beginning of the pandemic, testing for COVID was scarce in Denmark and reserved for people requiring hospitalization, so patients with mild or moderate symptoms just had to stay at home. In case these patients were suspected of having a disease other than COVID-19 (and depending on their condition), the GPs described that they had three potential choices: 1) postpone a face-to-face consultation until the respiratory symptoms vanished, 2) attempt to diagnose by video or telephone and prescribe medicine without physical examination, or 3) refer them to the hospital in a specific COVID testing path, including further examination for other diseases. When testing became more widely spread, most GPs would see the patients in the clinic if they could present a fresh, negative corona test.

In a few clinics, the GP or nurse conducted more home visits than usual, especially at the beginning of the corona period. The target group was especially elderly and fragile patients whom the GP wanted to protect from the risk and fear of getting infected in transport or in the clinic, and who did not have the opportunity to attend video consultations. Other GPs and nurses refrained from conducting home visits in order to avoid the risk of infecting the patient. Further, they conducted fewer visits to their patients in nursing homes, which were mainly replaced by telephone and video consultations with nursing home personnel.

*”We usually spend one afternoon a week at one of these nursing homes. And there was a period of time when it was purely on the telephone. And, that was, it's not as safe. Because do you get to see what you need to see then? But as soon as we have resumed, well from mid-May, I think, we started coming there again.” *(GP12, interview 2)

At the time of the first interview, the financial agreement for video and extended telephone consultations had just been made, and most of the GPs had had little experience using these consultations. Between the first and the second interviews, the use of video consultations first increased and later on decreased while the number of face-to-face consultations continuously increased again. At the time of our first interviews, the most common face-to-face consultations were with patients suspected of having cancer or heart problems or in need of minor surgeries. For some consultations (e.g. chronic care check-ups), the GPs chose to conduct the consultation in two parts: attendance at the clinic in order to perform tests, with a follow-up appointment by video or telephone. At the second interviews, most interviewees explained that they were quite close to a normal practice since they saw a predominant number of their patients face-to-face, reducing the need for video and telephone consultations.

There were differences both between the GPs and over time for individual GPs for which patients they used video, telephone and face-to-face consultations, and which health issues they experienced could be handled with these alternatives. Some GPs described that basically all their consultations were made face-to-face unless there were compelling reasons not to do so. Some GPs held almost all their consultations by video (also by the second interview), and the remaining GPs were somewhere in between. None of these differences could be linked to differences in the interviewees’ practice type, gender or seniority.

### Choice between face-to-face and video/telephone consultations

There was diverse reasoning behind the GPs’ choices between face-to-face or alternative consultations, but the main considerations were threefold: 1) *Minimization of risk; 2) Following guidelines; 3) A preference for face-to-face consultations.*

As for *minimization of risk,* the GPs considered both risk at the societal level, for selected patients, and for the personnel in the clinics. They tried to contribute to a reduction of the societal risk of infection by minimizing the number of patients in the clinic in general, and by compressing the time patients were there, for example, by only doing tests in person and continuing the consultation by telephone or video. They also focused on protecting more vulnerable patients by avoiding face-to-face consultations and by minimizing potential infection during transport, as well as in the clinic. Conversely, as one GP mentioned, it was important to ensure that chronically ill patients were well-regulated to minimize their risk of complications if they ever contracted COVID-19, and, therefore, some of them had to also be seen face-to-face. Considerations about the GPs’ and staff’s own risk was not a large issue during the interviews, but some considerations influenced their clinical activities and consultation choices, for example, they cancelled all spirometries, and one GP partner, who himself had an increased risk of COVID complications if infected, continued his use of video consultations longer than his colleagues. During the second interview, the GPs described how they had continuously followed and considered the development of the epidemic in Denmark, and that they had chosen to open up their clinics more and more as they judged it permissible due to a diminishing infection pressure and a general opening of society.

*“it was also related to an observation of the infection rate. Well, we could just see where it was going, and there were fewer and fewer who got it, and the infection pressure got better and better. So, on that basis, we thought, then we will also try to open up a little more.” *(GP11, interview 2)

Some also justified a more extensive use of face-to-face consultations with the physical opportunities in their clinics because they believed there was enough physical space to ensure an appropriate distance between patients.

As for *following guidelines*, the interviewed GPs had generally followed the guidance and list from DSAM, suggesting which health issue or diagnoses should be handled in face-to-face consultations or, alternatively, in other consultation forms. They saw the list as guidance, not absolute or sufficient in itself, since differential diagnoses, severity and other issues necessitated individual assessments.

*“Then again, there are also differential diagnoses and diagnostic considerations within the specific groups, even though it says it might be a green patient. Or a yellow patient. Or whatever it might be. So, we have also had to go in and say: ‘it does not make sense to see everyone just because they have a specific diagnosis’.” *(GP11, interview 1)

Overall, the interviewed GPs had a *preference for face-to-face consultations.* They chose this consultation form as soon as they judged it as being essential for ensuring sufficient patient care, especially if it did not jeopardize patient safety with the risk of infection or was not in conflict with the current guidelines. Further, some of the consultations that could not be meaningfully conducted without a physical examination were also carried out face-to-face to avoid a backlog of such consultations in the future.

*“So, we also strive to conduct some of the chronic care check-ups, also so that we do not get insanely behind and things somehow run wild.” *(GP6, interview 1)

Their preference for face-to-face consultations was also connected to the limitations they experienced with video and telephone consultations, which is elaborated upon in the next section.

The GPs generally mentioned that the video consultations had functioned well in specific areas, such as assessing children with fever, parts of chronic disease check-ups, follow-ups on test results, and medication adjustments (for which, in some cases, a telephone consultation was sufficient). Conversational therapy, musculoskeletal disorders, and assessing birthmarks for potential cancer were examples of issues that some GPs found could be assessed on video, while others insisted that face-to-face consultations were necessary. Some health issues were seen by the interviewees as acceptable to examine over video or by telephone given the extraordinary circumstances. They argued that they expected the COVID-related restrictions to last for a short period of time only, and that some issues could be assessed over video in a crisis situation, although not optimally.

*“They have pain in their shoulder, ‘can you lift it?’. Okay, there may be some things, but it also has its limitations because we cannot touch the patients. But in this crisis situation, it works really, really well.” *(GP13, interview 1)

The GPs were generally not worried that the altered and postponed consultations had consequences for the patients’ health. However, during the first interview, this position was linked to the expectation that the pandemic restrictions were expected to be for a limited time period only. By the second interview, this position was linked to the fact that the GPs had already increased their use of face-to-face consultations, and if a telephone or video consultation was insufficient, a visit in the clinic could be booked.

### Experienced differences between face-to-face and video/telephone consultations

#### Consultation content and form

Compared to face-to-face consultations, the GPs experienced that the video consultations were more focused on the issue at hand with fewer sidesteps, chitchat, and additional issues presented by the patients. They experienced both advantages and disadvantages using video consultations, which they weighed differently. On the one hand, video consultations were experienced as being advantageous because the consultation was more targeted, which ensured a better treatment.

*”Patients are good at limiting themselves; they have less of a tendency to elaborate and keep on talking. It's a great pleasure. Right to that point, it's actually really good.”* (GP8, interview 1)

On the other hand, video consultations were also perceived as being possibly disadvantageous, because the less focused talk between the patient and the GP often could lead the GP to uncover unspoken issues and important information on the patient’s both known and unknown health concerns. The GPs mainly found video consultations suitable for delimited and relatively simple issues. Further, an interviewee explained that one down-side of the concrete focus was that she became a quick-fix doctor instead of one being able to see things more holistically.

*“Then you can say the advantage in terms of time is that you limit yourself to one problem, where you in a consultation often are more holistic because there you go deeper into it. And you can always discuss, do you want to be a doctor extinguishing fires with a focus on one thing, or do you want to try to see everything as a whole. Therefore, video consultations are only suitable for very specific things.” *(GP2, interview 2)

### Relational and nonverbal limitations

Although nonverbal communication is possible to a larger degree over video compared to by telephone, it was experienced as problematically limited compared to face-to-face consultations. When using video, some GPs felt they lost sense of their patients and the space they shared with them since it was more difficult on screen than in person to read their patients’ nonverbal expressions, sense how they reacted, and watch their body language.

*”Well 90% of a consultation is nonverbal, and you miss it a bit when the interface is a screen. So it's more that the nonverbal communication is lost a bit, I think, and that can be a problem because it can be hard to figure out where I have the patient. So you have to be extra good at asking about worries, fears or expectations because it is harder to capture.” *(GP11, interview 2)

As some interviewees stated, they usually get an impression of a patient already when they pick them up in the waiting room, or when they enter the consultation room. The GPs found that some of the connection, presence and intimacy suffer when there is a screen between them and their patient. This is due to three main reasons: 1) interruptions from technical problems and other people being present in people’s homes during the consultation; 2) the GP and the patient do not have eye contact because one cannot simultaneously look at the camera and at the other person on the screen; and 3) the GP cannot lay a comforting hand on the patient in times of distress. For these reasons, several GPs found these consultations less appropriate in the case of existential crises and sensitive conversations.

*“It just seems like on a screen as if there is a distance. You do not get quite the same connection with the patient on a video. Just the fact that you do not really know where to look, where is the camera? And you look at two different places [if looking at the person in the screen, you do not look at the camera and hence have no eye contact]. And in that case, I think it is difficult. So, I do not really think that video is good for sensitive conversations.”* (GP10, interview 2)

### The clinical assessment and treatment

In relation to clinical assessments, the GPs described how limiting it can be not to be able to see and physically examine their patients nor take spontaneous tests during the consultation. However, increased diagnostic uncertainty was not a prominent issue. The GPs generally expressed that they felt they had a safety net because they could reschedule a consultation for a face-to-face follow-up in case of doubt.

*“But we also have a safety net under us in that we always say that if we, during the conversation, assess that it cannot be done this way, then the patient quickly gets an appointment for an assessment up here instead.” *(GP5, interview 2)

During the second interview, a couple of GPs described having experienced that their patients either returned more often after video consultations, or that they had to reschedule a large proportion of the video consultations into face-to-face consultations due to the insufficiency of the video consultation.

*"Video, it's actually almost gone again, and it's kind of surprising, but, uh, but it was not that easy to implement, and my impression was that more than half of the video consultations actually had to be followed up by face-to-face consultations because you could not clarify the problem on video” *(GP12, interview 2)

As a consequence of such experiences, some GPs chose to expand their use of face-to-face consultations again. They found face-to-face consultations more thorough, enabling the health issue at hand to be dealt with fully within only one consultation.

The GPs experienced that knowing the patient well was crucial when they had to change the usual consultations to video and telephone. When they knew their patients well, which they usually did, they also knew how each patient typically presented, and they felt able to see and hear whether everything looked and sounded as usual. They also found it easier to assess the level of urgency because they knew whether the patient usually contacted them with minimal issues or had a tendency to exaggerate the situation, or the opposite. They also knew which patients were capable of waiting and observing a potential worsening of a condition, and who were not. Hence, they felt that knowing the patient could compensate for some of the disadvantages of video and telephone consultations. In support of this understanding, one GP had experienced that video and telephone consultations were more difficult for GPs in training who did not know the patients. A few GPs even believed that when they knew the patient, telephone consultations worked just as well as video consultations for some issues. Lastly, in case of minor and delimited health issues, the GPs found this familiarity as being less crucial.

When not seeing the patients physically, and thereby not having the optimal basis for accurate clinical decision making, many of the GPs mentioned that their prescription of pharmaceuticals was a bit more liberal. Here, they generally mentioned antibiotics prescribed for COPD exacerbations, otitis media, sore throat, pneumonia symptoms, and sinusitis.

*“Everyone with respiratory symptoms, whether they are there because they are chronic ill patients, or they turn up because of the respiratory symptoms, they do not come up here, and we do not come out to them. There, we handle it on telephone. It also means that there are some for whom we under other circumstances would do a lot to avoid giving antibiotics, but to whom we right now by telephone end up having to say: ‘well we will have to try some penicillin’.” *(GP12, interview 1)

Similarly, because they could not perform a spirometry, patients suspected of having asthma were given medicine using a more trial and error approach. Further, unlike the usual practice, some GPs renewed prescriptions without having first seen the patient in the clinic, and some mentioned prescribing tranquilizers, morphine or other pain relievers on the basis of a telephone consultation, as well. However, under the given circumstances, this prescribing behaviour was considered better than first having a face-to-face consultation, which might increase the risk of spreading COVID-19.

### Technical limitations

The technical limitations involved both the *patients’ abilities and opportunities* and the *technical quality and disturbances.* The GPs experienced that some patients did not have the needed technical competencies or equipment. Some also had difficulties sitting correctly in front of the camera or handling it when they had to show things on their body. Here, the GPs also mentioned the work involved in informing patients about the technical issues before a video consultation. Further, the technical solution for video consultations was ill-suited for nursing homes. Some nursing homes lacked portable equipment that enabled them to bring the video consultation to the patient, and if the staff had to confer with the GP about several patients at the same time, the log-in to the video consultation was problematic because it could only be linked to an individual patient and not to the nursing home as a unit. Concerning the technical quality and disturbances, the GPs often experienced the quality of the pictures as being insufficient for diagnostic purposes due to the pixilation and limited sharpness of the pictures. Hence, some GPs asked their patients to send photographs instead. They also experienced technical disturbances in the form of poor internet connections and delayed or disappearing sound and picture during the consultations.

*“The quality of the video camera and the sharpness of the image, if you want to observe, for example a rash or swelling, it requires the patient being able to get the camera angled in the right place and that the light and the distance to what we want to see is okay […]. It is also preferable to use a Wi-Fi connection. A 3G network pixelates it and makes it blurry. It also means that some contrasts and sharpness are lost, and then you cannot really use it.” *(GP4, interview 1)

A GP described that even though he felt psychologically closer to the patient when using video compared to the telephone, the technical disturbances could influence the focus of their conversation and therefore make it less preferable. On the other hand, another GP found that some of the initial challenges had been overcome, and the videos were well-functioning when used.

### Choosing between video and telephone consultations

There were different reasons behind the GPs’ choosing between video and telephone consultations. Some mainly chose video consultations and only used telephone consultations when their patients were not able to use video, or because they had to switch a planned video consultation to the telephone due to technical problems. These GPs found it valuable to see the patients while talking to them, no matter the content of the consultation.

*"Video consultations are perhaps primarily for these other things, such as annual checks-ups, checks-ups for other things, where you have test results to consider, and some of it you can take over the phone, but personally I like to be able to see the person.” *(GP8, interview 1)

Other GPs only chose video if they needed to see the patient for clinical reasons, for example, to examine a rash, or to conduct conversational therapy where being able to observe facial expressions is important.

*“We only choose video when it will give us something extra, that is, where there is something we need to look at on the patient, a rash or movement, they need to point to the place where it hurts or a conversation about depression where you have to see what their face looks like and their facial expressions and that sort of thing. But otherwise, a lot is done on the phone, for sure.” *(GP5, interview 1)

These GPs perceived the telephone consultations to be as good as or preferred over the video consultations because it was easier when they only had to talk about data (e.g. test results), or because they did not believe it to be possible to diagnose the patients over video anyway. Here, some preferred a combination of telephone consultations and the patient sending in a photograph. With the possibility of having extended telephone consultations reimbursed, some stated that they actually realised how much can be done in this way. Hence, some GPs described that although they had anticipated they would use video consultations, they had increased their use of telephone consultations over video consultations.

## Discussion

In the initial phase of the pandemic, the GPs re-organised their usual clinical work profoundly. Most consultations were held over video or telephone, postponed or cancelled. Initially, the use of video and telephone consultations rose, but soon declined when the GPs deemed that face-to-face consultations were once again permissible. This decision was made both in relation to the infection pressure and the need to reopen society. When re-organising their practice, GPs considered how to minimize the risk of COVID and COVID complications, and they followed central guidelines and their own preference for face-to-face consultations. There were variations both over time and among the GPs regarding for which health issues video and telephone were used. Video, and in some cases the telephone, were found useful while there were restrictions on the use of face-to-face consultations, and these were generally found to be more suitable for simple and delimited issues. The GPs experienced some relational and technical limitations, whereas diagnostic uncertainty was not a prominent issue.

Data from the Danish general practitioners’ organisation on the national use of video and telephone consultations correspond to our findings [8]. The use of video initially rose, but peaked in the first week of April, and in mid-June, it was used minimally. Telephone consultations also rose and declined, and by mid-June, these were used more than videos. Face-to-face consultations increased correspondingly.

This study aligns with and expands the existing knowledge on alternative consultation forms. In previous studies on video consultations, similar challenges concerning the relational and technical issues were identified [9–12]. However, in these studies, the use of video consultations was examined in selected patient groups where it in advance was deemed potentially meaningful by doctors or researchers, often in intervention studies, instead of real-life, and in other settings than general practice [12]. Our real-life study provided knowledge of a more extensive use of video, involving a wider spread of GPs and health issues, and it demonstrated substantial, individual differences in what GPs experienced as feasible and acceptable when it comes to using alternative consultations forms under different conditions while still upholding adequate patient care.

In a recent study of video consultations during COVID in Spain, the GPs perceived such consultations as an adequate option, providing the benefit of reduced infection risk while having both verbal and non-verbal possibilities in comparison with telephone consultations. The potentially negative issues identified were again the technical, physical and relational limitations [13]. However, only a few of the participants had used this consultation form at the time of their data collection. Our study expands this knowledge because of the diverse use and experiences among the Danish GPs, and the changes over time with the altering pandemic conditions.

In their discussion paper, Bidmead and Marshall [14] consider how a profound use of video consultations during the pandemic can extend the current knowledge in this field, and they elaborate on the issues that need to be considered during their use. They state that, *“the crisis has provided a golden opportunity for large scale usage to be researched and for the findings of earlier research to be revisited”*. They question whether their experiences with video consultations during the pandemic might change the GPs’ reluctance towards video and its perceived limited usefulness, or whether clinicians will revert to their usual practice at the first opportunity [14]. In their paper, they highlight the importance of considering clinicians’ sense-making and buy-in [14]. Within implementation research sense-making (understanding usefulness and what to do differently) and buy-in (commitment in the new practice) are considered as being essential for ensuring a successful implementation [15]. Our study interestingly shows how the GPs initially found the alternative consultation forms meaningful for a range of health issues (and hence gained experiences here) and had a faster buy-in than in most usual implementation processes. However, both their sense-making and buy-in were situationally conditioned, and their willingness to use these alternatives generally diminished when face-to-face consultations were deemed viable.

Our study suggests that the GPs’ weighing of the benefits and limitations of alternative consultation forms is context-bound and therefore varies with changing conditions, options and challenges, which again influence their willingness to use these alternatives. Whereas limitations seem less dependent on the context (though technical limitations might become fewer over time and with experience), the weighing up of these limitations with the potential benefits and need for alternative consultations might differ. The context influencing both their weighing-up of the limitations and willingness to use alternative consultations in this study can be attributed to four contributing factors: the changing infection pressure and the associated assessment of the viability of face-to-face consultations; the safety net option (the possibility of converting to a face-to-face consultation); the belief that the pandemic would have a limited time span; and the challenges when conducting video consultations. Their choice of whether to use video or telephone was also influenced by situational circumstances. Although some found new possibilities by using extended telephone consultations, it seemed that the telephone was primarily a viable alternative to video either because of technical challenges with the video consultations or because the GPs already saw most of their patients face-to-face. Diagnostic uncertainty was not a prominent issue in this study. A study from the early phase of the pandemic found that Flemish GPs’ who used telephone consultations as their primary source of contact feared missing a diagnosis because they had to rely on their patients’ descriptions and self-examinations [16]. Once again, it was an important contextual condition in the Danish setting that the GPs could always reschedule a video or telephone consultation into a face-to-face consultation if deemed necessary.

In other settings and in non-pandemic times, other contextual circumstances, such as geographical distances, technical limitations, the types of patients etc., might influence the weighing up of the benefits and limitations and hence willingness to use these alternatives. Moreover, as we demonstrated, willingness is also influenced by individual GP preferences and presumably also patient preferences (which we have not explored). Hence, our study shows the importance of incorporating contextual conditions and preferences when considering and planning for alternative consultation forms.

### Strengths and limitations

This study provides insights into the reorganization of consultations in the first months of the pandemic. It is a strength that we interviewed the GPs twice, which ensured deeper insights into both the initial and changing practices and experiences as the pandemic developed. However, due to the continuously changing pandemic and a re-rise in infection rates after our data collection, a third round of interviews could have been additionally enlightening. Eleven of the GPs provided daily notes for our research project, and these notes gave us a good impression supplementary to the interviews of how daily work in the clinic was affected by the pandemic; for example, how the GPs experienced the development in the guidelines, testing capacity, and the use of video consultations. Further, our study broadens the knowledge on the use of alternative consultation forms as it depicts a real life setting and an unselected group of GPs who have not initiated their use due to a special interest or participation in a research study.

## Conclusion

In the initial months of the pandemic, GPs re-organized their clinical work. They first converted a vast number of consultations to video or telephone, or postponed or cancelled them, and later on increasingly resumed their usual practice. They were guided by their intention to minimise the risk of COVID, the national guidelines, and their own preference for face-to-face consultations. Video, and in some cases the telephone, were found to be useful when there were restrictions on the use of face-to-face consultations. The GPs experienced the same kinds of limitations and drawbacks as have been previously found in non-pandemic times, which influenced their willingness to use these alternatives. The weighing of pros and cons and their willingness to use these options shifted and generally diminished as soon as face-to-face consultations were deemed viable. This reduced buy-in is worth taking into account in non-pandemic times if considering a more general roll-out and implementation of video or telephone consultations in general practice. However, in future pandemics or other situations with a need for radical changes of medical work, these alternatives seem valuable, at least for a limited period of time, when seeking to uphold adequate patient care in general practice.

## Data Availability

The anonymised transcribed interviews from the current study are available from the corresponding author upon reasonable request.

## Abbreviations

GP: General practitioner
DSAM: Dansk Selskab for Almen Medicin [The Danish College of General Practitioners]
